# Editorial: Consequences of sleep deprivation

**DOI:** 10.3389/fnins.2023.1254248

**Published:** 2023-08-01

**Authors:** Andrea Romigi, Ritchie Edward Brown

**Affiliations:** ^1^IRCCS Neuromed Istituto Neurologico Mediterraneo Pozzilli (IS), Pozzilli, Italy; ^2^Universita Telematica Internazionale UNINETTUNO, Rome, Italy; ^3^Laboratory of Neuroscience, VA Boston Healthcare System and Harvard Medical School, Department of Psychiatry, West Roxbury, MA, United States; ^4^Boston VA Research Institute, Boston, MA, United States

**Keywords:** sleep deprivation, 24/7 society, sleep restriction, sleep recovery, cognition

Sleep deprivation occurs when an individual consistently fails to obtain an adequate amount of sleep, either due to external factors or internal disruptions. Sleep deprivation is a pervasive problem that affects millions of people worldwide. In our fast-paced and demanding modern society, where productivity is often prioritized over rest, the value of a good night's sleep has been overlooked and downplayed. Karen Russell, the acclaimed author of the dystopian novel “*Sleep donation*”, envisaged a near future with hundreds of thousands of cases suffering from an terminal insomnia crisis, where sleep is a commodity which can be donated to the lucky few (Russell, 2014). While the ability to donate sleep to others appears far-fetched at present, it is clear in the scientific community that the consequences of sleep deprivation can be far-reaching, impacting not only our physical health but also our mental wellbeing and cognitive abilities. Thus, this Research Topic which focuses on the mechanisms, consequences and biomarkers of sleep deprivation is important and timely and can inform the development of novel brain stimulation or pharmacological treatments to prevent accidents and ameliorate neuropsychiatric disorders involving sleep disruption (Brown et al., 2022). For instance, findings that increased orexin levels in patients Alzheimer's disease are linked to both sleep disruption and cognitive impairment (Liguori et al., 2014) suggest that the use of orexin receptor antagonists could be beneficial.

Sleep disruption affects virtually all physiological functions. Roach et al., using sophisticated molecular/genetic tools in a Drosophila model, identified specific “clock” neurons that are modulated by sleep disruption to change temperature preference. Interestingly, while sleep deprivation, sleep fragmentation and a social jetlag protocol led to a change in temperature preference, only the sleep deprivation protocol impaired memory formation suggesting that temperature preference is a more sensitive indicator of sleep disruption than learning and memory and a potential biomarker. The relationship between sleep and memory was evident from the 1st century when the orator Quintilian stated ≪…*quae statim referri non poterant, contexuntur postera die, confirmatque memoriam idem illud tempus quod esse in causa solet oblivionis…*≫, “What may seem unattainable initially can be effortlessly achieved the following day, and the time that is commonly thought to induce forgetfulness (i.e., sleep) is discovered to enhance memory” (Quintilian). Two millennia later, in 1924, Jenkins and Dallenback documented the first experimental evidence of the sleep effect preventing the normal memory decay curve (Jenkins and Dallenbach, 1924). Whitney et al. revised narratively the generally accepted approach to analyzing effects of sleep deprivation on subsequent memory and learning by means of its effects on encoding. The authors suggested an intriguing framework with which to understand sleep loss and memory in terms of temporary amnesia from sleep loss (TASL). The view of the TASL framework is that amnesia and the amnesia-like deficits observed during sleep deprivation not only affect memory processes but will also be apparent in cognitive processes that rely on those memory processes, such as decision-making. The hippocampus, interacting with higher structures, such as the prefrontal cortex, to produce complex cognition and behavioral performance, is compromised by sleep disruption. Li B. et al. used resting-state functional magnetic resonance imaging (fMRI) to study the relationship between the changes of the precuneus (PC) functional connectivity and alertness decline after total sleep deprivation (SD). SD induced decreased functional connectivity between the right PC and the right middle frontal gyrus (MFG). Moreover, there was a significant correlation between the decreased PC functional connectivity and alertness decline after total SD. They hypothesized that the interruption of the connection between the right PC and the right MFG is related to the observed decline in alert attention after acute SD. These results provide further indications of the changes in cortical circuits which underlie cognitive impairments during SD. Functional connectivity changes also occur in primary insomnia. Xie et al. examined the abnormal resting state functional connections (RSFCs) in patients with insomnia. The authors found structural changes in the right middle frontal gyrus and right inferior frontal gyrus accompanied by RSFC changes. Thus, these brain regions may represent potential targets for non-invasive brain stimulation treatments (Brown et al., 2022). Continuing the cognitive theme Wu et al., evaluated neurocognitive disorders including postoperative delirium and cognitive decline in the early postoperative period in patients with or without excessive daytime sleepiness who had a moderate to high risk of obstructive sleep apnea (OSA). The authors found a significant correlation with postoperative cognitive decline for those OSA patients who had excessive daytime sleepiness, a potentially treatable phenotype.

Two studies by Peng and colleagues investigated the effect of total SD (TSD) on evoked potentials and working memory. The first study from Peng, Dai et al. found that TSD can impair working memory capacity, which is characterized by lower amplitude and prolonged latency of working-memory related N2-P3 ERPs components. In a second study Peng, Hou et al. utilized ERPs to investigate the restorative effects of 8 h of recovery sleep on working memory impairments induced by total sleep deprivation for 36 h. Eight hours of recovery sleep attenuated the decrease in working memory performance caused by 36 h of TSD. The authors hypothesized that these restorative effects are likely to occur during SWS. However, RS had limited effects and further studies should establish whether 8 h of RS can restore cognitive function to baseline levels.

In our 24/7 society a generally held presumption has been that while chronic sleep disruption results in neurobehavioral impairments, performance deficits are reversed with limited-period recovery sleep (e.g., over the weekend) (Zamore and Veasey, 2022). Li C. et al., studied the effects of 52 h of SD and of 14 h of recovery sleep on resting-state fMRI and the availability of A1 adenosine receptors using a PET scan. The authors found negative correlations between A1AR availability and BOLD activity in the left superior/middle temporal gyrus and left postcentral gyrus of the human brain providing new insights into the molecular basis of neuronal responses induced by high homeostatic sleep pressure. The relationship between sleep and pain is complex and bidirectional. Sleep deprivation can exacerbate low back pain by lowering the pain threshold and increasing sensitivity to pain (Alsaadi et al., 2014), Luo et al. showed in an analysis of a large GWAS dataset that there was a mutual causal relationship between genetic variants which affect sleep and low back pain, therefore sleep regulators should be considered in a more comprehensive management of pain, if this observation will confirmed by prospective studies. Alterations of sleep continuity negatively affect synaptic plasticity in the hippocampus (Tartar et al., 2006) but less attention has been directed to the effects of sleep disruption in other parts of the nervous system. Han et al. evaluated how sleep deprivation (SD) affects the process of olfactory sensory neurons (OSN) regeneration following olfactory epithelium injury. SD accompanied by disturbed circadian activity could induce structurally negative effects on OSN regeneration, preferentially in the dorsomedial area of the OE, and that this area-specific regeneration delay might involve the biological activity of neurons expressing NQO1 (quinone dehydrogenase 1). Therefore, circadian activity modulates adult neurogenesis supporting the hypothesis of relationship with neurodegenerative diseases as well as olfactory disorders.

Increasing evidence suggests a role for the immune system and inflammatory responses in both normal sleep regulation and disrupted sleep (Irwin, 2019). The review by Amini et al. investigates whether there is a causal relationship between SD and the NLRP3 inflammasome, a key component of the innate immune responses, or its downstream pathways. They conclude that indeed the NLRP3 inflammasome is a potential target for therapy in order to improve the clinical outcomes of SD.

The 24/7 society is convinced that sleep will become increasingly more flexible and reduced sleep time will allow for greater personal freedom and success, until there is no difference between day and night, light and dark, and action and repose (Crary, 2013). This point of view is contrary to the findings of sleep and circadian science, which show that sleep disruption is detrimental to productivity increases accidents and impairs both physical and mental health (Czeisler, 2006). The 12 articles in this Research Topic illustrate the breadth of research on this topic, revealing the effects of SD on attention, working memory, temperature preference, pain and neural plasticity and some of the potential molecular mediators and brain circuits which are affected. Furthermore, they suggest that treatment of sleep disruption could have beneficial effects in the treatment of post-operative cognitive disorders and pain. We hope the readers will find them useful and interesting.

## Author contributions

All authors listed have made a substantial, direct, and intellectual contribution to the work and approved it for publication.
